# Green biosynthesized silver nanoparticles using aqueous extract of Salix alba: antimicrobial and cytogenetic effects on mitosis

**DOI:** 10.12688/f1000research.172513.1

**Published:** 2026-03-02

**Authors:** Worood Kamil Shalash Al Maliky, Jalal Hasan Mohammed, Mohamednoruldin Dh Hazim

**Affiliations:** 1 Biology Department, College of Education for Pure Sciences (Ibn Al-Haitham), University of Baghdad, Baghdad, Iraq; 2 College of pharmacy, University of Karbala, Karbala, Karbala Governorate, Iraq; 3 College of Biotechnology, Medical and Molecular Department, Al-Nahrain University, Baghdad, Iraq

**Keywords:** Salix alba, plant extract, silver nanoparticles (NPs), antimicrobial activity, cytogenetic assay, mitotic index

## Abstract

**Background:**

Green synthesis of silver nanoparticles (AgNPs) using plant extracts has gained increasing attention as an environmentally friendly alternative to conventional chemical methods. *Salix alba* contains bioactive phytochemicals that can act as reducing and stabilizing agents during nanoparticle synthesis. This study aimed to synthesize AgNPs using an aqueous bark extract of *S. alba* and to evaluate their antimicrobial and cytogenetic effects.

**Methods:**

Silver nanoparticles were synthesized by mixing *S. alba* aqueous extract with silver nitrate solution. Nanoparticle formation and surface characteristics were assessed using ultraviolet visible spectroscopy, atomic force microscopy (AFM), and scanning electron microscopy (SEM). Antimicrobial activity against *Escherichia coli*, *Staphylococcus aureus*, and *Candida albicans* were evaluated using the agar well diffusion method. Cytogenetic effects were assessed in mice bone marrow cells by measuring the mitotic index.

**Results:**

AFM and SEM analyses showed that AgNP size and morphology varied with silver nitrate concentration, with smaller and more uniform nanoparticles observed at lower concentrations. The synthesized AgNPs exhibited antimicrobial activity against all tested microorganisms, with inhibition zones increasing in a dose dependent manner. Cytogenetic analysis demonstrated a significant reduction in mitotic index in AgNP treated groups at higher concentrations, while *S. alba* extract alone did not induce genotoxic effects and maintained normal or slightly elevated mitotic index values.

**Conclusions:**

*Salix alba* mediated silver nanoparticles demonstrated antimicrobial activity and concentration dependent on cytogenetic effects. These findings indicate the importance of careful dose optimization when considering biologically synthesized AgNPs for potential applications.

## 1. Introduction

Nanotechnology is a rapidly growing field that brings together concepts from physics, chemistry, biology, and materials science to address various challenges in medicine, industry, and the environment ( Das *et al*., 2025). Among the many types of nanomaterials, silver nanoparticles (AgNPs) have received particular attention due to their strong antimicrobial effects, catalytic properties, and promising roles in medical and diagnostic applications ( Sati *et al*., 2025). However, traditional methods for producing AgNPs often rely on harmful chemicals and require large amounts of energy, raising serious concerns about their environmental impact and long-term sustainability. As an alternative, researchers have turned to green synthesis approaches that make use of biological sources such as plant extracts, bacteria, fungi, and algae ( Kirubakaran *et al*., 2025). These eco-friendly techniques not only minimize toxicity and environmental harm but are also more affordable and compatible with biological systems ( Shahzadi *et al*., 2025). So, The use of plant extracts in the eco-friendly synthesis of silver nanoparticles (AgNPs) has become increasingly favoured, primarily due to the presence of diverse bioactive compounds such as flavonoids, polyphenols, tannins, and terpenoids. These phytochemicals serve essential functions in the synthesis process, acting as natural reducing and stabilizing agents that facilitate nanoparticle formation ( Aji *et al*., 2025). A notable example is *S. alba* (commonly known as white willow), a tree native to many parts of Europe, Asia, and North America. Traditionally recognized for its therapeutic potential, *S. alba* contains several important phytochemicals; such as salicin, catechins, gallic acid, and other phenolic compounds—that are responsible for its antioxidant, antimicrobial, and anti-inflammatory activities ( Chaudhary *et al*., 2025a). These naturally occurring substances not only help in transforming silver ions into nanoparticles but also act as a protective layer on the nanoparticles surface, enhancing both their stability and biological functionality ( Lasmi *et al*., 2025).

In recent years, *S. alba* has gained attention as a promising natural source for synthesizing silver nanoparticles. Studies have demonstrated that aqueous extracts derived from the bark or leaves of *S. alba* can effectively mediate the formation of silver nanoparticles, typically characterized by their small size, spherical shape, and notable surface plasmon resonance (SPR) properties ( Habibi *et al*., 2025). These green-synthesized AgNPs have exhibited significant antimicrobial effects against a broad spectrum of pathogens, including both Gram-positive and Gram-negative bacteria, as well as various fungal strains. The antimicrobial action of these nanoparticles operates through multiple pathways, such as compromising microbial cell membranes, inducing reactive oxygen species (ROS), hindering DNA replication, and inhibiting vital enzymes ( Ibrahim *et al*., 2025). Moreover, the phytochemicals present in *S. alba* extracts may enhance the biological effectiveness of the nanoparticles by forming a stabilizing organic layer, which not only boosts antimicrobial activity but may also help reduce toxicity toward human cells ( Al-shattrawi, 2025).

In addition to their antimicrobial properties, the cytogenetic effects of silver nanoparticles (AgNPs) have become a growing focus of research, especially regarding their potential to cause genetic damage. One of the standard methods for evaluating genotoxicity is the micronucleus (MN) assay, which identifies small extranuclear structures that result from chromosomal fragments or entire chromosomes that are not properly integrated into daughter nuclei during cell division ( Lala, 2025). The presence of micronuclei is considered a dependable marker for detecting chromosomal instability and genotoxic stress in both cell cultures and living organisms ( Onoja *et al*., 2025). As AgNPs are increasingly explored for biomedical applications, it becomes essential to assess not just their therapeutic potential but also any cytogenetic hazards they may pose ( Bentrad, 2025). Green-synthesized AgNPs, which are coated with plant-based biomolecules, may offer a safer alternative with lower genotoxicity compared to nanoparticles produced through conventional chemical methods. Nonetheless, their actual cytogenetic impact is influenced by several variables, such as particle size, surface charge, dosage, and duration of exposure ( Zhang *et al*., 2025).

## 2. Methods

### 2.1 Plant collection and pretreatment

In September 2024, leaves of *S. alba* plant were gathered locally from Baghdad marketplaces. The plant was identified by Dr. Ibrahim S. Al–Jubouri/College of Pharmacy /Al–Mustansiriyah University/Iraq.

### 2.2 Preparation of the aqueous extract

The aqueous plant extract of *S. alba* was prepared using the traditional method ( Ghazali *et al*., 2022) by washing the plant parts with D.W. well to remove contaminants from the surface and drying them well with dry air for three days. A 50 g of willow bark were ground well and placed in a glass beaker with a capacity of 500 mL. that contains 300 mL of D.W. Then the extract was filtered using Whatman No. 1 filter paper and stored at 4°C for later use.

### 2.3 Reagents and chemicals used

All chemicals used in this study were of analytical grade. Silver nitrate (AgNO _3_, Sigma-Aldrich, Cat. No. S7653), potassium chloride (KCl, Sigma-Aldrich, Cat. No. P9541), and phosphate-buffered saline (PBS, Gibco, Cat. No. 10010023) were employed during nanoparticle synthesis and experimental procedures. Giemsa stain used for cytogenetic evaluation was obtained from Merck (Cat. No. 1.09204). All reagents were prepared according to the manufacturers’ instructions, and the amounts used in each experiment are detailed within the methodological subsections.

### 2.4 Biosynthesis of silver nanoparticles

Silver nanoparticles (AgNPs) were synthesized according to Subhani *et al*., (2024) using willow bark of *S. alba* aqueous extract as a plant-based reducing agent and silver nitrate (AgNO _3_) as the silver source. The reaction mixtures were prepared by combining 9 mL of AgNO _3_ at varying concentrations (1.0, 1.5, 2.0, and 2.5 mM) with 1 mL of the previously prepared willow bark extract. The mixtures were then incubated in a dark chamber at 30 °C for 24 hours without agitation to prevent photoreduction of silver nitrate. Each experiment was performed in triplicate (n = 3) to ensure reproducibility. pH was maintained at 7.0, and visual color change was monitored at regular intervals (every 2 hours) until stabilization.

### 2.5 Characterization of the plant-based green synthesized of silver nanoparticles

The subsequent methods were used to characterize the plant-based green synthesized nanoparticles. Every test administered in the laboratory of Ministry of Science and Technology, Baghdad, Iraq.

**2.5.1 Visual observation**

Silver nanoparticles are characterized by a noticeable color change by different period, which serves as an important indicator for the early detection of green-synthesized NPs.

**2.5.2 Ultraviolet visible is spectroscopy**

One practical approach for identifying the formation of green-synthesized nanoparticles by ( Masood *et al*., 2025) through UV–Vis spectrophotometry, where a distinct absorption peak typically appears around 433 nm, indicating the presence of surface plasmon resonance associated with the nanoparticles.

**2.5.3 Atomic force microscopy (AFM) and scanning electron microscopy (SEM)**

Atomic force microscopy (AFM) and scanning electron microscopy (SEM) analyses were conducted using an NT-MDT scanning probe microscope. The nanoparticle samples were first diluted with distilled water, then a drop of the diluted solution was placed on a clean glass slide (1×1 cm). After allowing the sample to air-dry completely, the slide was mounted on the AFM sample stage, and imaging was performed following standard operating procedures ( Tran *et al*., 2025).

**2.5.4 Antimicrobial activity**

The bacterial sample that used in this study was provided by the Molecular Biology Laboratory for postgraduate research/Department of Biology/College of Science/University of Baghdad.

**2.5.5 Antimicrobial activity of silver nanoparticles**

Mueller Hinton agar was prepared by dissolving 38 g of the medium in 1 L of distilled water. The solution was heated until completely dissolved and sterilized by autoclaving at 121 °C for 15 min. After cooling to 45–50 °C, the medium was poured into sterile Petri dishes (20–25 mm in depth) under aseptic conditions and allowed to solidify. For antimicrobial testing, two bacterial strains ( *Staphylococcus* aureus and *Escherichia coli*) and one fungal strain ( *Candida albicans*) were used. A single colony from each strain was inoculated into brain–heart infusion broth and incubated for 18 h at 37 °C. After incubation, 1 mL of each culture was transferred into 5 mL of sterile saline and vortexed to achieve homogeneity. The turbidity of the suspensions was adjusted to match 0.5 McFarland standard (~1.5 × 10 ^8^ CFU/mL). By using sterile swabs, the standardized microbial suspensions were evenly spread across the entire surface of the agar plates to ensure uniform distribution. Then, 100 μL of green-synthesized AgNPs from *S.* *alba* extract were carefully applied to the surface using a sterile micropipette. Four concentrations of AgNPs (100, 75, 50, and 25 mg/mL) were tested. The inculated plates were incubated at 37 °C for 18 h, after which antimicrobial activity was evaluated based on the diameter of inhibition zones formed around each application site.

**2.5.6 Animal acclimatization and experimental design**

Adult male Swiss Albino mice (Mus musculus), aged between 8–10 weeks and weighing 23–27 g, were obtained from the Biotechnology Research Centre at Al-Nahrain University. The animals were housed under standard laboratory conditions for acclimatization. The animals were randomly assigned into three groups, each consisting of Six mice: (1) Control group: Received normal saline. (2) AgNO3 NP group: received a single dose of 150 mg/kg. (3) *Salix alba* extract group: a single dose of 400 mg/kg. Following treatment, all animals were sacrificed and femoral bone marrow was extracted to calculate the mitotic index.

**2.5.7 Euthanasia and anaesthesia procedures**

All procedures involving animals, including anesthesia and euthanasia, were performed in accordance with the American Veterinary Medical Association (AVMA) Guidelines for the Euthanasia of Animals (2020). Prior to euthanasia, mice were briefly anesthetized using isoflurane inhalation (2–3% in oxygen) to minimize distress. Euthanasia was performed by cervical dislocation while under anesthesia, as recommended for small laboratory rodents. The dose of colchicine (0.25 mL of 1 mg/mL, intraperitoneal) used for metaphase arrest was applied following standard cytogenetic protocols and did not cause pain or distress at the time of euthanasia.

**2.5.8 Chromosome preparation of the mouse bone marrow**

The experiment was done according to ( Allen *et al*., 1977) as follow: For cytogenetic preparation, each animal was intraperitoneally injected with 0.25 ml of colchicine (1 mg/ml) to arrest cells in metaphase. Following euthanasia by cervical dislocation, the femur was aseptically extracted, and the bone marrow was flushed using 5 ml of phosphate-buffered saline (PBS, pH 7.2) into a sterile test tube. The suspension was centrifuged at 2000 rpm for 10 min., and the supernatant was discarded. Cells were then treated with 5 ml of 0.075 M potassium chloride (KCl) as a hypotonic solution and incubated at 37°C with intermittent shaking. After a second centrifugation at the same speed and duration, a chilled fixative was added dropwise with gentle agitation to a final volume of 5 ml. The mixture was kept at 4°C for 30 min. and centrifuged multiple times to ensure proper fixation, with the final suspension adjusted to 2 ml of fixative. A few drops (4–5) were dropped vertically from a height of approximately 90 cm onto pre-chilled glass slides to ensure proper chromosome spreading. After air drying, the slides were stained with Giemsa solution for 15 min. and rinsed with distilled water. Three slides were prepared per animal for cytogenetic examination under the microscope. This section must include enough detail on the data sources and processes so that others can reproduce your research.

### 2.6 Ethics approval

All animal procedures in this study were reviewed and approved by the Animal Ethics Committee of Al-Nahrain University, College of Biotechnology. The study was conducted in full compliance with institutional guidelines for laboratory animal care and the American Veterinary Medical Association (AVMA) Guidelines for the Euthanasia of Animals (2020). No additional permits or animal licenses were required for this study; if this information is not available, this is because the institution does not issue separate license numbers for routine academic research involving mice.

### 2.7 Statistical analysis

The results were statistically analysed using one-way ANOVA followed by Tukey’s post-hoc test to compare differences between groups. A significance level of p < 0.05 was considered statistically significant. All analyses were carried out using SPSS version 25.0 (IBM Corp., Armonk, NY, USA).

## 3. Results and discussion

### 3.1 Biogenic silver nanoparticles

**Figure 1.**
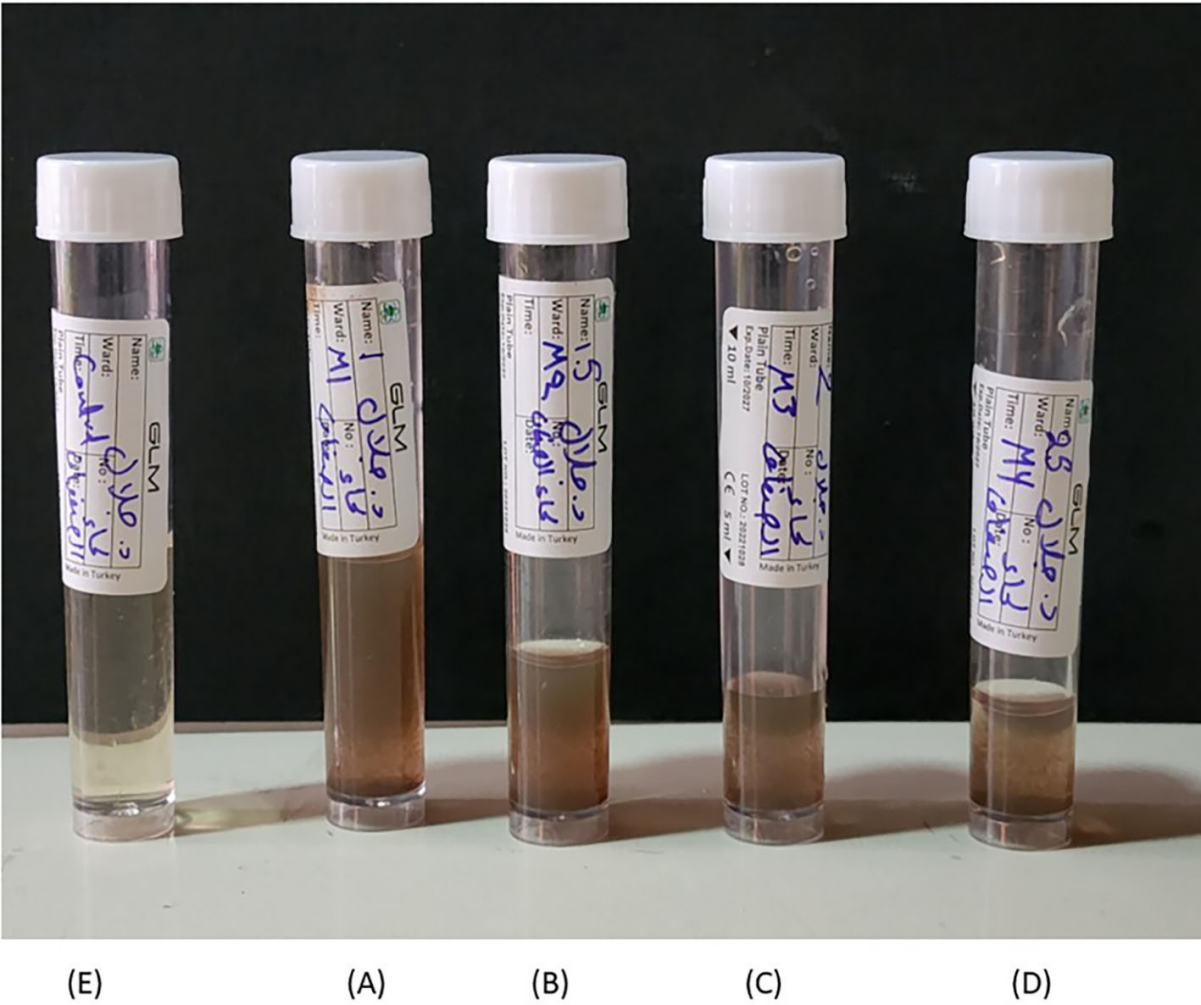
Visual observation of green-synthesized silver nanoparticles after 24 hours. (A–D) represent willow bark extract loaded with silver nanoparticles at concentrations of 1, 1.5, 2, and 2.5 mM AgNO _3_, respectively. (E) shows the aqueous willow bark extract without the addition of AgNO _3_.

The formation of green silver nanoparticles was confirmed through noticeable changes in the color of the reaction mixture as well as spectrophotometric analysis ( Hussain *et al*., 2025). Initially, the solution began to change from yellow to light brown within the first 10 minutes of mixing ( Figure 1), and this color gradually intensified to dark brown after 24 hours of incubation. This shift in color indicates the successful reduction of silver ions (Ag ^+^) into elemental silver nanoparticles (Ag ^0^), likely driven by the active phytochemicals present in the willow bark extract. The brown coloration is commonly linked to the excitation of surface plasmon resonance (SPR), which is characteristic of silver nanoparticles ( Muhammad *et al*., 2025). Spectral analysis showed a distinct absorbance peak around 433 nm, confirming the presence of AgNPs. The intensity of this SPR peak increased with higher concentrations of AgNO _3_, reaching its maximum at 2 mM, suggesting more efficient nanoparticle formation at this concentration. The synthesis process may have been influenced by hydrophilic and hydrophobic interactions among the components, which can promote nanoparticle stability and shape ( Hindryawati *et al*., 2025).

**Figure 2.**
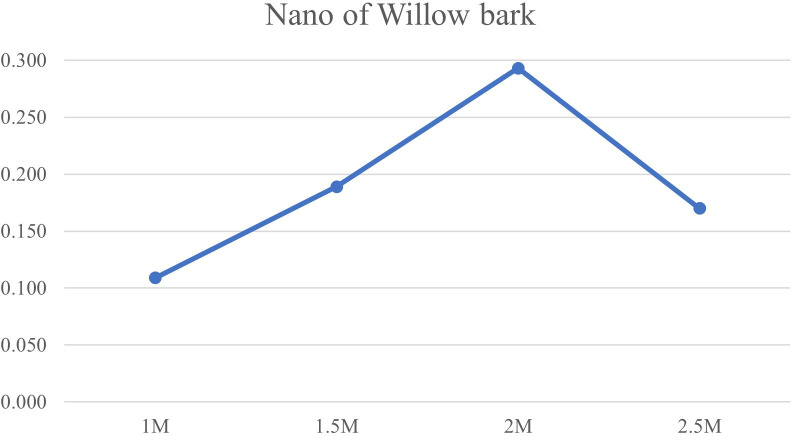
UV–visible absorption spectra of green-synthesized silver nanoparticles, showing a characteristic surface plasmon resonance peak at 433 nm.

These findings are in line with previous reports showing that silver nanoparticles in aqueous solution typically exhibit brown coloration due to SPR excitation ( Khalid *et al*., 2025). Additionally, factors such as the composition of the biological extract and the concentration of metal salts play a key role in determining nanoparticle yield and characteristics. The unique optical properties of nobale metals like silver stem from their ability to support surface SPR, making them ideal for nanoparticle-based applications ( Saini *et al*., 2025) ( Figure 2).

**Figure 3.**
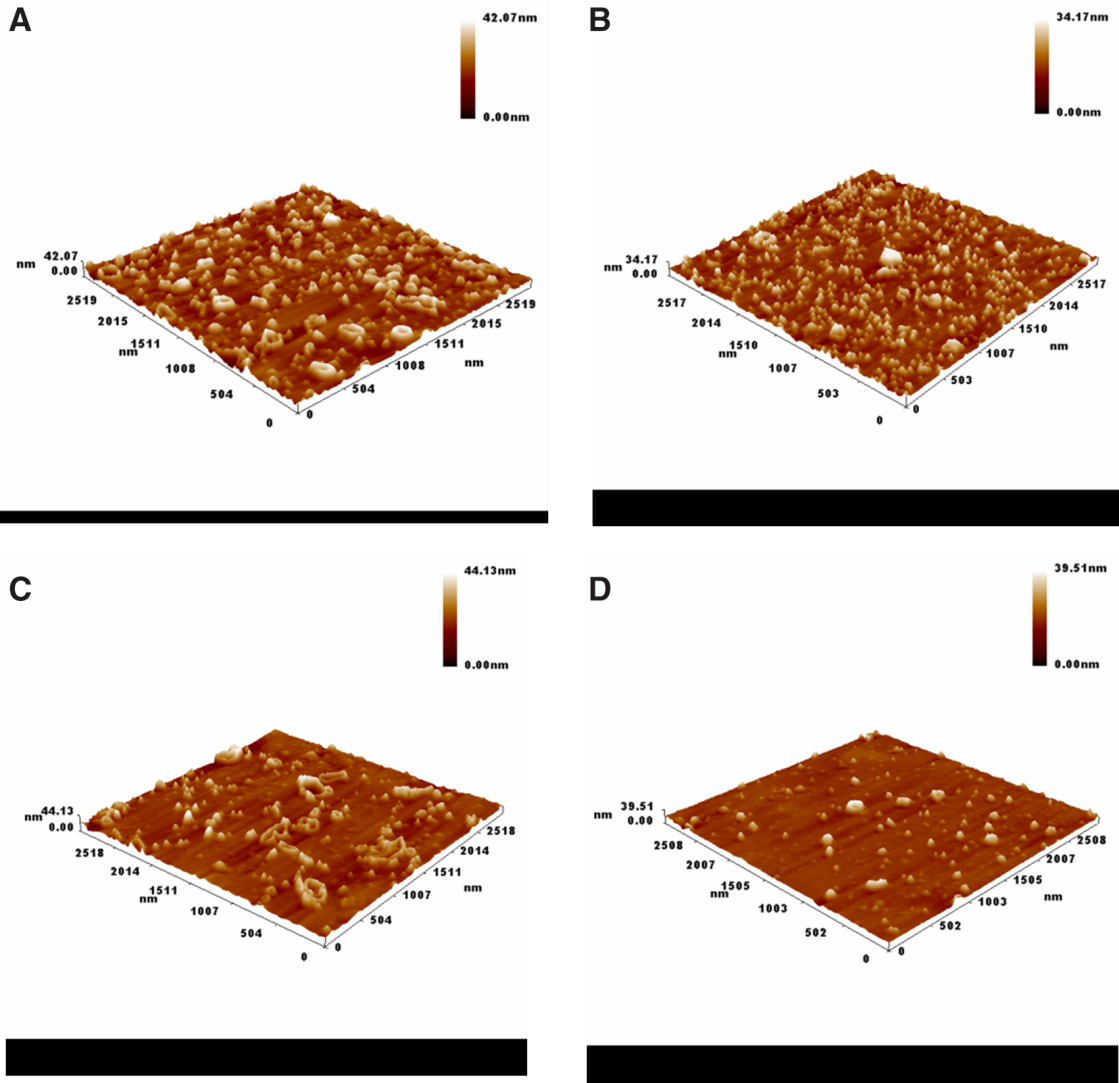
Atomic Force Microscopy (AFM) 3D surface morphology of green-synthesized silver nanoparticles at different AgNO _3_ concentrations: (A) 1 mM, (B) 1.5 mM, (C) 2 mM, and (D) 2.5 mM.

Atomic force microscopy (AFM) imaging provided valuable insight into the surface morphology and topography of the green-synthesized silver nanoparticles at varying silver nitrate concentrations. As seen in ( Figure 3 A–D), the nanoparticles exhibited distinguishable differences in size distribution and surface roughness depending on the precursor concentration.

At 1 mM ( Figure 3-A), the nanoparticles were densely distributed with an average particle size of approximately 79.57 nm and an average height of 75 nm, indicating a relatively uniform structure that similarity to Jilani *et al*., (2025).

As the concentration increased to 1.5 mM ( Figure 3-B), the average size decreased to 69.19 nm with a reduced height of 65 nm. This suggests that a moderate increase in AgNO _3_ concentration may enhance the nucleation rate, yielding smaller and more compact nanoparticles due to limited aggregation ( Koçer and Özçimen 2025).

Interestingly, at 2 mM ( Figure 3-C), the average particle size rose to 85.66 nm and the height reached 80 nm. This might be attributed to particle growth dominating over nucleation, likely caused by the saturation of reducing agents in the *S. alba* extract, resulting in fewer but larger particles ( Benaissa *et al*., 2025).

However, when the concentration increased to 2.5 mM ( Figure 3-D), the particle size again decreased to around 70 nm with a height of 60 nm. This fluctuation indicates that beyond a certain concentration threshold, the stabilizing capacity of the phytochemicals becomes insufficient to control the growth, leading to partial aggregation and size variation ( Elumalai *et al*., 2025).

**Figure 4.**
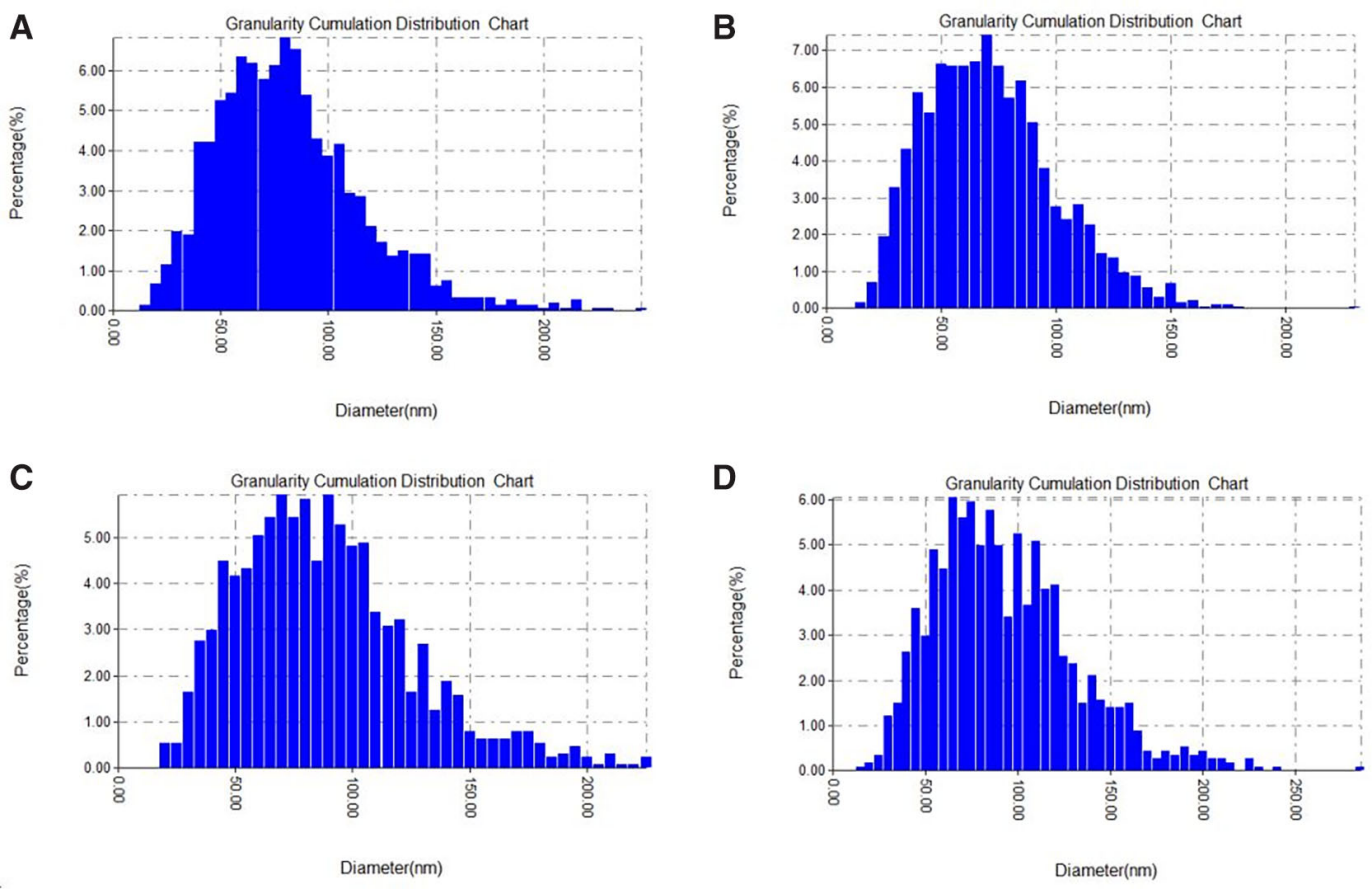
AFM particle size distribution analysis of green-synthesized silver nanoparticles at different AgNO _3_ concentrations: (A) average height of 75 nm and 79.57 nm for average size, (B) average height of 65 nm and 69.16 nm for average size, (C) average height of 80 nm and 85.60 nm for average size, and (D) average height of 60 nm and 70 nm for average size.

The grain size distribution charts ( Figure 4 A-D), further support these observations, showing a shift in peak particle populations depending on the concentration. Most particles fell within the 50–100 nm range, consistent with effective nanoscale synthesis. The narrow size distribution observed, especially at 1.5 and 2.5 mM, suggests enhanced control over particle growth when silver ion concentration and phytochemical reducing agents are well balanced ( Zhu *et al*., 2025).

Overall, the AFM results confirm that silver nitrate concentration plays a crucial role in determining nanoparticle size, height, and distribution. Optimal synthesis appears to occur around 1.5 to 2 mM, where particle uniformity and stability are most favorable. These findings highlight the sensitivity of green synthesis to reactant concentrations and the importance of fine-tuning reaction parameters to achieve desirable nanoparticle characteristics ( Wilson *et al*., 2025).

As the previous figure which that presents AFM-based measurements of the surface heights of green-synthesized silver nanoparticles prepared at varing silver nitrate concentrations. The recorded average heights were 75 nm (1 mM), 65 nm (1.5 mM), 80 nm (2 mM), and 60 nm (2.5 mM), indicating noticeable morphological variation. These differences suggest that the concentration of silver precursor plays a key role in controlling nanoparticle growth and surface topology.

**Figure 5.**
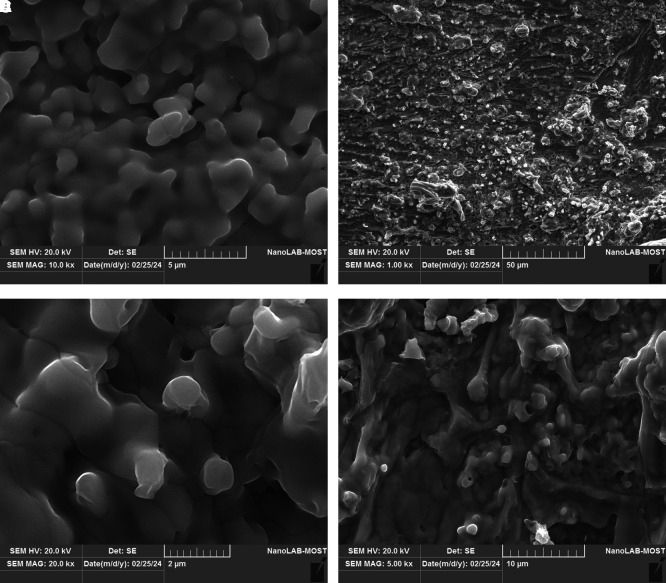
Scanning electron microscopy (SEM) images of green-synthesized silver nanoparticles at different silver nitrate concentrations: (A) 1 mM with an average particle size of approximately 5 μm, (B) 1.5 mM with an average particle size of approximately 50 μm, (C) 2 mM with an average particle size of approximately 2 μm, and (D) 2.5 mM with an average particle size of approximately 2 μm.

The SEM images as Figure 5 A–D reveal clear differences in surface morphology and particle aggregation of green-synthesized silver nanoparticles at various magnifications. Spherical to semi-spherical particles are visible with varying levels of distribution, indicating successful formation and surface interaction influenced by silver nitrate concentration ( Ahmed and Rahmah, 2025).

### 3.2 Antimicrobial activity

**Figure 6.**
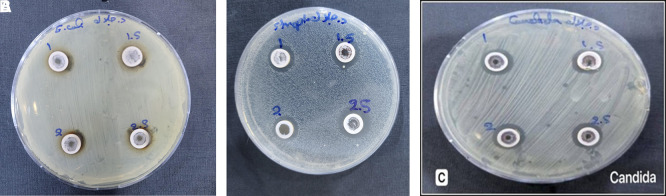
Antibacterial activity of silver nanoparticles using aqueous extract of *Salix alba* at different molarities (1, 1.5, 2, and 2.5 mM) against (A) *Escherichia coli*, (B) *Staphylococcus aureus*, and (C) *Candida albicans.*

The experimental part of the study included several biological assays to evaluate the antimicrobial activity of the prepared extract against selected bacterial ( *E. coli* and *S. aureus*) and fungal strains ( *C. albicans*) under laboratory conditions, as shown in Figure 6 A-C.

In this study, the aqueous extract of S. alba was used to synthesize silver nanoparticles and tested for antimicrobial activity against *E. coli in vitro* ( Figure 6-A). Petri dishes containing nutrient medium were treated with different extract concentrations (1.0, 1.5, 2.0, and 2.5 mM). The inhibition zones measured 8, 10, 14, and 15 mm, respectively. The gradual increase in inhibition zone diameter with higher concentrations confirms a dose-dependent antibacterial effect of the synthesized nanoparticles ( Agrawal *et al*., 2025).

Where, the antibacterial activity of the *S. alba* based silver nanoparticle extract was evaluated against S. aureus using a nutrient-rich medium inoculated with the bacterial strain. Different extract concentrations (1, 1.5, 2, and 2.5 mM) produced inhibition zones measuring 13, 15, 17, and 18 mm, respectively. Previous studies which Turay (2025) who estimated that the gradual increase in zone diameter with higher concentrations confirms a positive correlation between extract dose and antibacterial effectiveness. Although, Shalaby *et al*., (2025). The gradual increase in zone diameter with higher concentrations confirms a positive correlation between extract dose and antibacterial effectiveness. This enhanced activity at elevated concentrations may be attributed to the increased availability of silver nanoparticles and phytochemicals, which together intensify cell wall disruption, protein denaturation, and oxidative stress within the bacterial cells ( Wang *et al*., 2025), as shown in Figure 6-B.

Furthermore, the antifungal activity of the green-synthesized AgNO _3_ NPs was evaluated against *C. albicans.* As shown in the Figure 6-C, four different concentrations of the S. alba NPs extract that have different molarity concentration (1, 1.5, 2, and 2.5 mM) were tested. Clear inhibition zones were observed around each well (13, 15, 17 and 18 mm), respectively, with their diameters increasing proportionally with concentration ( Ibrahim *et al*., 2025). This suggests a dose-dependent antifungal effect, likely due to enhanced NPs interaction with the fungal cell membrane. The increased presence of bioactive compounds and silver ions may contribute to membrane disruption, oxidative stress, and enzyme inhibition in C. albicans ( Beyatli, 2025).

When compared with other plant-mediated silver nanoparticles, the antimicrobial performance of *S. alba* appears to be equally strong or superior. For example, *Cassia siamea*-derived AgNPs produced inhibition zones of 10–14 mm *against S. aureus* ( Turay, 2025), while *Acacia mangium*-based AgNPs showed zones of 11–15 mm against *E. coli* ( Aji *et al*., 2025). Similarly, *Xanthium strumarium*-mediated nanoparticles demonstrated inhibition zones around 12–15 mm against *C. albicans* ( Beyatli, 2025). In our study, *S. alba*-derived AgNPs exhibited larger inhibition zones (up to 18 mm against *S. aureus* and *C. albicans*), suggesting that the phytochemical composition of willow bark rich in salicin, flavonoids, and phenolic compounds may enhance both nanoparticle stability and biological activity. These comparative results highlight the distinctive contribution of *S. alba* among plant-based nanoparticle systems.

The antimicrobial and cytogenetic effects of AgNPs are generally belong to a combination of physical, chemical, and biochemical mechanisms rather than a single pathway. At the microbial level, AgNPs can adhere to the negatively charged bacterial cell wall, damaging membrane integrity and increasing permeability, which cause leakage of essential ions and metabolites ( Ibrahim *et al*., 2025). Once internalized, AgNPs release silver ions (Ag ^+^), which interact with sulfur- and phosphorus-containing biomolecules such as proteins and DNA, thereby impairing enzymatic activity and replication processes ( Beyatli, 2025). Another well-documented mechanism is the generation of reactive oxygen species (ROS), including superoxide anions, hydroxyl radicals, and hydrogen peroxide, which induce oxidative stress, damage lipids, oxidize proteins, and fragment nucleic acids ( Wang *et al*., 2025).

### 3.3 Chromosome from somatic cells of the mouse bone marrow

**Figure 7.**
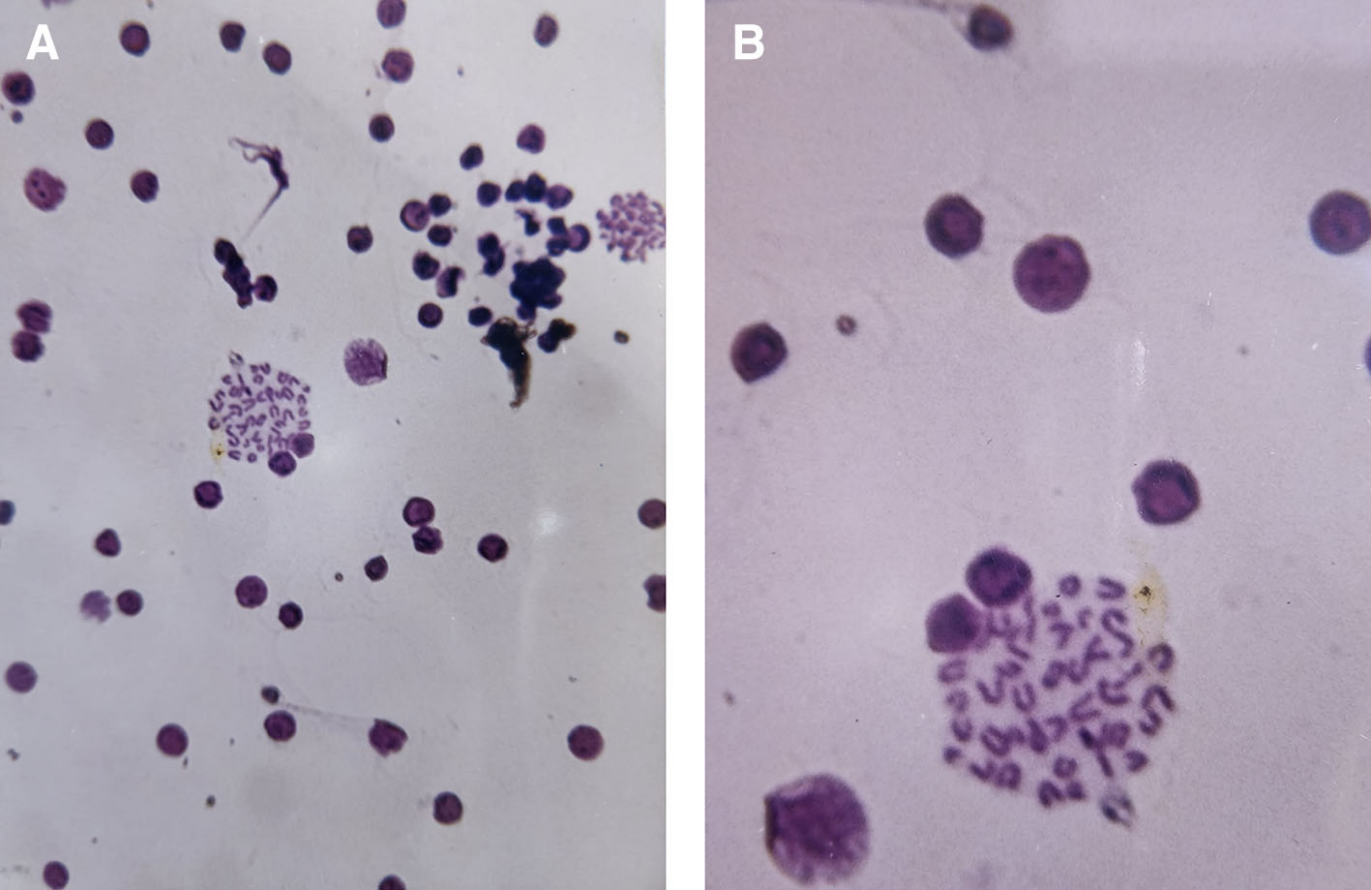
Cytogenetic effects of aqueous extract from *Salix alba* in mice bone marrow cells. (A) Representative microscopic image showing a general field view of bone marrow cells. (B) Magnified view highlighting mitotic cells.

The results clearly indicated a decrease in mitotic index in silver nanoparticles treated group as compared with negative control. While it increased in plant extract group to 7.8% as compared to negative group 5%. As shown in Table 1 and Figure 7 (A-B).

**Table 1.** Mitotic index (%) values of bone marrow cells in mice treated with different concentrations of biosynthesized silver nanoparticles compared with the control group.

Groups	Mitotic index % (M±SE)	P value
Negative control	5.0 ± 0.30 ^a^	p < 0.001 → ***
Single dose of AgNO _3_ NPs (150 mg/kg)	1.2 ± 0.14 ^b^	p < 0.001 → ***
Plant extract group (400 mg/kg of *Salix alba*)	7.8 ± 0.14 ^c^	p < 0.001 → ***

*** Indicates a highly statistically significant difference (p < 0.001). Different superscript letters (a, b, c) indicate statistically significant differences between groups according to the post hoc multiple comparison test, where groups with different letters differ significantly at p < 0.05.

At the cytogenetic level, AgNPs may directly interact with chromosomes and mitotic machinery. Several studies have demonstrated that AgNPs can cause chromosomal aberrations, spindle disruption and disruption of cell-cycle progression, which is consistent with the observed reduction in mitotic index ( Tang, 2025; Osman *et al*., 2025). For cancer therapy for the recent decades natural products play a important role in the development of a drug. In addition to the possible relationship between natural products and regulation of telomerase working ( Shalash *et al*., 2021). ROS-mediated DNA strand breaks, coupled with impaired repair pathways, further contribute to chromosomal instability ( Zhang *et al*., 2025). Additionally, AgNPs have been reported to interfere with mitochondrial respiration, leading to ATP depletion and triggering apoptosis, which indirectly reduces the pool of actively dividing cells ( Bentrad, 2025).

It is also worth noting that the biological response to AgNPs is influenced by multiple factors, including nanoparticle size, shape, surface charge, and the phytochemical composition of the reducing agent used in synthesis ( Kirubakaran *et al*., 2025). The phytochemicals in *S. alba* extract such as flavonoids, tannins, and salicin are likely to play a important role: (i) reducing and stabilizing silver nanoparticles, and (ii) exerting protective antioxidant effects that mitigate genotoxicity. This may explain why the group treated with *S. alba* extract alone showed normal or elevated mitotic index, in contrast to the significant suppression observed in the AgNP-treated groups. Such duality highlights the importance of considering both nanoparticle characteristics and plant-derived biomolecules when evaluating biosafety as explained in ( Chaudhary *et al*., 2025b).

Overall, the results demonstrate that *S. alba* mediated AgNPs exhibit strong antimicrobial and measurable cytogenetic effects in a dose-dependent manner. To better interpret these outcomes, that compare our findings with previous studies and discuss possible mechanisms that may explain the observed biological activities.

## 4. Conclusion

This study demonstrates the successful green synthesis of AgNPs using aqueous *S. alba* bark extract, confirmed by visual color change, UV–Vis spectroscopy, AFM, and SEM analyses. The biosynthesized AgNPs exhibited significant antimicrobial activity against *E. coli*, *S. aureus*, and *C. albicans*, with inhibition zones increasing in a concentration-dependent manner. Additionally, cytogenetic evaluation indicated that while chemically synthesized AgNPs reduced the mitotic index, the *S. alba* extract maintained or enhanced mitotic activity, suggesting a protective biological effect. These findings highlight the dual potential *of S. alba* based AgNPs as both antimicrobial and biocompatible agents, offering a promising eco-friendly alternative for biomedical applications.

## Data Availability

The raw data supporting the findings of this study are publicly available in the Figshare repository at https://doi.org/10.6084/m9.figshare.30944873 ( AlMaliky, 2025a). The dataset includes raw atomic force microscopy (AFM) images and corresponding instrument output reports, raw antimicrobial inhibition zone measurements against *Escherichia coli*, *Staphylococcus aureus*, and *Candida albicans*, and raw cytogenetic mitotic index data. The data are openly accessible with no embargo or login requirement and are available under the terms of the Creative Commons Attribution 4.0 International (CC BY 4.0) license. The completed ARRIVE 2.0 checklist for reporting animal research is also publicly available on Figshare at https://doi.org/10.6084/m9.figshare.31046287 ( AlMaliky, 2025b). Extended data supporting this study are available in a public repository. The extended data includes raw atomic force microscopy (AFM) images and instrument output reports, raw antimicrobial inhibition zone measurements against Escherichia coli, Staphylococcus aureus, and Candida albicans, and raw cytogenetic mitotic index data.
