# Supplementary material for: Green biosynthesized silver nanoparticles using aqueous extract of Salix alba: antimicrobial and cytogenetic effects on mitosis
Source: F1000Res. 2026 Jun 3;15:331. Originally published 2026 Mar 2. [Version 2] doi: 10.12688/f1000research.172513.2 (PMC13241840; doi:10.12688/f1000research.172513.2)
Supplement: Peer review history

# Reviewer report: Reviewer response for version 2

**DOI:** 10.5256/f1000research.198859.r490720

**Published:** 2026-06-05

**Authors:** Santosh Mallikarjun Bhavi, Akshata Choudhari Padti

**Affiliations:** 1 Applied Genetics, Karnatak University Dharwad, Dharwad, Karnataka, India

The authors have comprehensively addressed all comments and suggestions raised during the review process. The manuscript has been substantially improved, and the revised version is now suitable for indexing.

Is the work clearly and accurately presented and does it cite the current literature?

Partly

If applicable, is the statistical analysis and its interpretation appropriate?

Partly

Are all the source data underlying the results available to ensure full reproducibility?

Partly

Is the study design appropriate and is the work technically sound?

Partly

Are the conclusions drawn adequately supported by the results?

Partly

Are sufficient details of methods and analysis provided to allow replication by others?

Partly

Reviewer Expertise:

Green synthesis of nanoparticles, nanoparticle characterization, and biological applications.

We confirm that we have read this submission and believe that we have an appropriate level of expertise to confirm that it is of an acceptable scientific standard.

# Reviewer report: Reviewer response for version 2

**DOI:** 10.5256/f1000research.198859.r490719

**Published:** 2026-06-05

**Authors:** Nourhane A. Darwich

**Affiliations:** 1 Biological Science, Beirut Arab University, Beirut, Lebanon

The authors have adequately addressed all reviewer comments and implemented the required revisions. The manuscript has been significantly improved in terms of clarity, presentation, and scientific quality. Therefore, I recommend the paper for indexing.

Is the work clearly and accurately presented and does it cite the current literature?

Yes

If applicable, is the statistical analysis and its interpretation appropriate?

Partly

Are all the source data underlying the results available to ensure full reproducibility?

Yes

Is the study design appropriate and is the work technically sound?

Partly

Are the conclusions drawn adequately supported by the results?

Yes

Are sufficient details of methods and analysis provided to allow replication by others?

Yes

Reviewer Expertise:

Green synthesis of nanoparticles; nanobiotechnology; in vitro and in vivo experimental models; nanotoxicology; oxidative stress and antioxidant assays; antimicrobial activity; antidiabetic studies; nanoparticle characterization (UV–Vis, FTIR, XRD, TEM, SEM, XPS, DLS); histopathology and biological evaluation of nanomaterials; molecular docking and computational analysis.

I confirm that I have read this submission and believe that I have an appropriate level of expertise to confirm that it is of an acceptable scientific standard.

# Reviewer report: Reviewer response for version 1

**DOI:** 10.5256/f1000research.190246.r470711

**Published:** 2026-04-01

**Authors:** Nourhane A. Darwich

**Affiliations:** 1 Biological Science, Beirut Arab University, Beirut, Lebanon

This manuscript presents an interesting study on the green synthesis of silver nanoparticles using *Salix alba* extract and evaluates their antimicrobial and cytogenetic effects. The topic is relevant and aligns with current trends in sustainable nanotechnology. However, several methodological and interpretative aspects require clarification to improve the scientific rigor and reproducibility of the study.

**Question to author**

  

1. Could the authors clarify the rationale behind selecting the specific AgNO₃ concentrations (1–2.5 mM)?
2. Were optimization studies (pH, temperature, reaction time) performed before selecting the final synthesis conditions?
3. How was batch-to-batch reproducibility of nanoparticle synthesis ensured? \
4. The study relies mainly on UV–Vis, AFM, and SEM. Why were DLS, zeta potential, or XRD not included to confirm size distribution, stability, and crystallinity?
5. The UV–Vis peak at ~433 nm confirms nanoparticle formation, but can the authors provide full spectra and peak width analysis for better interpretation?
6. SEM images (Figure 5) report particle sizes in micrometers, which contradict nanoscale findings from AFM. Could the authors clarify this discrepancy?
7. Is there evidence of nanoparticle aggregation in SEM images, and how might this affect biological activity?
8. Why was only the agar well diffusion method used instead of more quantitative methods such as MIC or MBC?
9. Were positive controls (e.g., standard antibiotics) included for comparison?
10. How was the concentration of AgNPs (mg/mL) calculated, given that synthesis was based on molarity (mM)?
11. The study includes only three experimental groups. Why was a nanoparticle dose-response design not included for cytogenetic analysis?
12. The dose of AgNPs (150 mg/kg) appears relatively high—what is the justification for this dose selection?
13. Could the authors explain how many cells were counted per slide for mitotic index calculation?
14. The manuscript mentions ANOVA with Tukey’s test. Were assumptions (normality, homogeneity of variance) verified before analysis?
15. Why are standard deviations or confidence intervals not consistently reported alongside mean values?
16. Some conclusions suggest “biocompatibility,” yet cytogenetic results show reduced mitotic index. Can the authors better reconcile this apparent contradiction?
17. The discussion largely describes results; could the authors provide deeper mechanistic insight supported by recent literature?
18. How do these findings compare quantitatively with other plant-mediated AgNPs reported in recent studies?
19. Several grammatical and typographical errors are present throughout the manuscript. Careful language editing is recommended.
20. Figures require improved clarity, labeling, and consistency (e.g., units, magnification, scale bars).
21. Some sections of the methods lack sufficient detail to ensure reproducibility (e.g., nanoparticle purification steps).
22. I recommend that the authors incorporate additional relevant references to strengthen the discussion, particularly regarding nanoparticle characterization techniques and the antibacterial activity of silver nanoparticles
  Refer to reference 1&2

Is the work clearly and accurately presented and does it cite the current literature?

Yes

If applicable, is the statistical analysis and its interpretation appropriate?

Partly

Are all the source data underlying the results available to ensure full reproducibility?

Yes

Is the study design appropriate and is the work technically sound?

Partly

Are the conclusions drawn adequately supported by the results?

Yes

Are sufficient details of methods and analysis provided to allow replication by others?

Yes

Reviewer Expertise:

Green synthesis of nanoparticles; nanobiotechnology; in vitro and in vivo experimental models; nanotoxicology; oxidative stress and antioxidant assays; antimicrobial activity; antidiabetic studies; nanoparticle characterization (UV–Vis, FTIR, XRD, TEM, SEM, XPS, DLS); histopathology and biological evaluation of nanomaterials; molecular docking and computational analysis.

I confirm that I have read this submission and believe that I have an appropriate level of expertise to confirm that it is of an acceptable scientific standard, however I have significant reservations, as outlined above.

## References

[1] : Silver and Yttrium-Doped Silver Nanoparticles From Pine Needle Leaf Extract: Synthesis, Characterization, Antioxidant, Antiuropathogenic Bacterial, and Docking Activities. *Bioinorganic Chemistry and Applications, 2025(1), 1566870* .2025;

[2] : Green Synthesis of Silver and Silver Oxide Nanoparticles from Cedrus Libani: Biological Activity and Influence of Film Thickness on Optical Properties. *Ukrainian Journal of Physical Optics, 27(1), 01080–01101* .2026;

# Reviewer report: Reviewer response for version 1

**DOI:** 10.5256/f1000research.190246.r470719

**Published:** 2026-03-30

**Authors:** Santosh Mallikarjun Bhavi

**Affiliations:** 1 Applied Genetics, Karnatak University Dharwad, Dharwad, Karnataka, India

The manuscript presents an interesting study on the green synthesis of silver nanoparticles using Salix alba bark extract and their antimicrobial and cytogenetic effects. The topic is relevant and has potential applications in nanobiotechnology. However, several issues related to methodological clarity, citation accuracy, data presentation, and overall consistency need to be addressed before the manuscript can be considered for indexing. The following comments are provided to help improve the quality and clarity of the work.

1. Authors are requested to mention the complete scientific name of the plant at first mention; is it Salix alba L.? Please confirm and maintain consistency throughout the manuscript.
2. In Section 2.1, authors mentioned collection of leaves, whereas throughout the study bark extract is used. Authors need to clarify whether leaves or bark were actually used, as this creates confusion and affects reproducibility.
3. In the Introduction, the sentence “Traditionally recognized for its therapeutic potential…”—the citation needs to be changed or the sentence needs to be revised, as the cited paper discusses the Salix genus, whereas the sentence specifically refers to S. alba. Kindly update it accordingly.
4. In the Introduction, the statement regarding synthesis of silver nanoparticles cites a paper that appears to be about gold nanoparticles. Authors are requested to cross-check this and all other citations carefully for similar issues.
5. Is the bark sample heated during preparation of the aqueous extract? If yes, mention the temperature and time in Section 2.2.
6. In Section 2.4, the phrase “willow bark of S. alba” is slightly redundant and can be improved for clarity and scientific precision. Consider revising it to “Salix alba bark” or “bark of Salix alba”.
7. In Section 2.5.2, do not include discussion or expected results such as the peak at 433 nm in the Methods section. This should be moved to the Results and Discussion section. Also, include the scanning range of the UV–Vis spectrophotometer and the instrument details.
8. If possible, authors are encouraged to perform XRD for crystalline nature, FTIR for functional group analysis, and EDX for elemental composition for better characterization.
9. Authors should clearly mention which method was used for antimicrobial activity (agar well diffusion or disc diffusion). Why was a standard antibiotic not used as a control in the antimicrobial study? This is necessary. Also, the concentration of AgNPs used (25–100 mg/mL) seems too high. Please check whether this is a typographical error (µg instead of mg). Furthermore, the study is mentioned as dose-dependent, but Figure 6 shows different molarity of synthesized AgNPs rather than different concentrations of AgNPs. Authors need to clarify this.
10. There is also a conceptual confusion between AgNO₃ molarity used during synthesis and AgNP concentration used in biological assays. Authors should clearly distinguish between these.
11. In Figure 1, the authors have shown one image of AgNO₃ and another image after mixing AgNO₃ with bark extract (for different molarities). However, it would be clearer if the authors include an image of the reaction mixture immediately after adding the bark extract to AgNO₃ (time zero) and another image of the same mixture after a certain incubation time, showing the color change due to bioreduction. This would better demonstrate the nanoparticle formation process.
12. In Figure 2, authors should plot the complete UV–Vis spectrum for all four molarity-based synthesized AgNPs, not only at 433 nm. Please use standard software such as Origin. Also, change the title from “Nano of the willow bark” to “UV–visible spectroscopy”.
13. Why did authors use different scale bars for SEM images (e.g., 5 µm, 50 µm, 2 µm, and 10 µm)? This creates confusion and makes comparison difficult. Since four different types of AgNPs were synthesized (based on different AgNO₃ molarities), authors should present SEM images of all four samples using a common scale bar for proper comparison in the main manuscript. If needed, images with different magnifications can be included in the supplementary data.
14. For chromosomal study, authors mentioned "single dose of AgNO₃NPs (150mg/kg)".Is this referring to AgNPs? If yes, which molarity-based synthesized sample was used? Also, Figure 7 shows only extract-treated cells; what about AgNP-treated cells?
15. Authors should clearly describe how mitotic index was calculated, including the number of cells counted and the criteria used.
16. The rationale for selecting the dose (150 mg/kg) is not mentioned; authors should justify this.
17. Some conclusions, such as claims about safety or biocompatibility, seem overstated based on the presented data and should be moderated.

Is the work clearly and accurately presented and does it cite the current literature?

Partly

If applicable, is the statistical analysis and its interpretation appropriate?

Partly

Are all the source data underlying the results available to ensure full reproducibility?

Partly

Is the study design appropriate and is the work technically sound?

Partly

Are the conclusions drawn adequately supported by the results?

Partly

Are sufficient details of methods and analysis provided to allow replication by others?

Partly

Reviewer Expertise:

Green synthesis of nanoparticles, nanoparticle characterization, and biological applications.

I confirm that I have read this submission and believe that I have an appropriate level of expertise to confirm that it is of an acceptable scientific standard, however I have significant reservations, as outlined above.

# Response

**Published:** 2026-04-14

**Authors:** Worood Al Maliky

**Affiliations:** College of Education, University of Baghdad College of Education for Pure Science Ibn Al-Haitham, Baghdad, Baghdad Governorate, Iraq

we sincerely thank the reviewer for the positive evaluation and constructive comments. All suggestions have carefully considered and addressed in the revised manuscript.

 1. Thank you for your valuable comment. The complete scientific name of the plant is Salix alba L. This has been verified and is now correctly stated at the first mention, with consistent usage maintained throughout the manuscript

 2.We would like to clarify that the reference to “leaves” in Section 2.1 was a typographical error. The study was performed using the bark extract of Salix alba L.. This has been carefully revised and corrected throughout the manuscript to avoid any confusion and to ensure reproducibility.

 3.The sentence has been revised to correctly reflect that the cited reference refers to the Salix genus rather than specifically to Salix alba L.. The text has been updated accordingly to ensure accuracy and consistency.

 4.We have carefully reviewed the cited references and confirmed that references [4] and [5] are directly relevant to the synthesis of silver nanoparticles. Reference [3], which discusses nanoparticles in general, has been retained only where appropriate for general statements. All citations have been revised to ensure accuracy and proper alignment with the text.

 5.The extraction procedure has been clarified in Section 2.2, including the temperature (60 °C) and extraction duration (30 minutes), to ensure reproducibility of the method

 6.The phrase has been revised to “Salix alba bark” to improve clarity.

 7.The description of the UV–Vis analysis has been revised by removing result-related statements and including the instrument model (SmartSpec™ 3000, Bio-Rad Laboratories, USA) and scanning range (200–800 nm) in Section 2.5.2.

 8.Thank you for your insightful suggestion. We agree that XRD, FTIR, and EDX analyses would provide additional information regarding crystallinity, functional groups, and elemental composition. However, due to limited access to these advanced characterization facilities, these analyses could not be performed in the current study. Nonetheless, nanoparticle formation was adequately confirmed through UV–Vis spectroscopy and supported by AFM and SEM analyses.

 Future studies will include these analyses for further validation.

 9.The antimicrobial method has been clarified as the agar well diffusion method in Section 2.5.4.

 Regarding the absence of a standard antibiotic control, we acknowledge its importance. However, the primary aim of this study was to investigate the relative antimicrobial activity of the synthesized AgNPs. This limitation has now been addressed and discussed in the revised manuscript.

 Concerning the reported concentrations (25–100 mg/mL), we appreciate the reviewer’s observation. This was indeed a typographical error, and the correct unit is µg/mL, which has now been corrected throughout the manuscript.

 Finally, we agree with the reviewer regarding the terminology used. The antimicrobial activity presented in Figure 6 corresponds to different molarity-based synthesized AgNPs, rather than different applied doses. Therefore, the term “dose-dependent” has been revised to “concentration-dependent” to accurately reflect the experimental design.

 10.Thank you for this important and insightful comment. We agree that there was a lack of clarity in distinguishing between the molarity of AgNO₃ used during nanoparticle synthesis and the concentration of AgNPs used in the biological assays.

 In the original version, the values expressed in mM were mistakenly described as “extract concentrations,” which may have caused confusion. These values in fact refer to the AgNO₃ precursor molarity used during the synthesis of AgNPs, not to the concentration of AgNPs in the antimicrobial assay.

 The manuscript has now been carefully revised to clearly distinguish between these two parameters. All relevant sections have been corrected by replacing terms such as “extract concentrations” and “concentration-dependent” with more accurate expressions, including “AgNPs synthesized from different AgNO₃ precursor molarities.”

 11. In response, we have included additional images to better illustrate the nanoparticle formation process. Specifically, images of the reaction mixture were added at:

 0 h (immediately after mixing), and

 2 h after incubation,

 The observed variation in color intensity over time suggests dynamic changes in nanoparticle formation, aggregation, and stabilization processes. Image have been uploaded as revised figure files and data availability

 12. Thank you for this valuable suggestion. We agree that presenting the complete UV–Vis spectra for all synthesized AgNP samples would provide a more comprehensive characterization of the nanoparticles.

 However, the UV–Vis analysis was conducted in a collaborating laboratory outside the country, and due to current logistical constraints and limited access to the original instrumental data, it is not possible at this stage to retrieve or reprocess the full spectral datasets.

 Nevertheless, the characteristic surface plasmon resonance (SPR) peak at 433 nm has been reported, which is widely recognized as a reliable indicator of AgNP formation. The manuscript has been revised to clarify this point.

 In addition, the figure title has been corrected from “Nano of the willow bark” to “UV–visible spectroscopy” as recommended

 13.In response, the SEM images of all four AgNP samples (corresponding to different AgNO₃ molarities) have been revised and are now presented using a uniform scale bar to allow proper comparison, as recommended.

 The updated SEM images have been uploaded as revised figure files and linked to the corresponding figure (Figure 5) in the manuscript.

 Additional images at different magnifications remain available in the Data Availability section

 14.The term “AgNO₃NPs” has been corrected to “AgNPs” throughout the manuscript. The chromosomal study was conducted using three experimental groups: a negative control group, an AgNP-treated group (150 mg/kg body weight), and an extract-treated group (150 mg/kg body weight). The in vivo study was designed as a dose-based evaluation rather than a comparison between different AgNO₃ molarity-based synthesized samples.

 Additionally, Figure 7 has been revised and clarified to indicate the respective treatment groups.

 No noticeable morphological differences were observed in mitotic cell appearance among the control, AgNP-treated, and extract-treated groups. The effects of the treatments were primarily quantitative, as reflected by changes in the mitotic index, rather than qualitative changes in mitotic morphology.

 15.The mitotic index (MI) was calculated based on metaphase-arrested cells due to the use of colchicine, which inhibits spindle fiber formation and arrests cells at the metaphase stage.

 Therefore, only cells observed in metaphase were considered as dividing cells. A total of 1000 cells were examined under a light microscope (40×), and the MI was calculated using the standard formula:

 MI (%) = (Number of metaphase cells / Total number of cells counted) × 100.

 This has now been clarified in the revised manuscript.

 The mitotic index (MI) was determined by counting metaphase-arrested cells following colchicine treatment. Only cells observed in metaphase were considered as dividing cells.

 This approach is commonly used in cytogenetic studies to enhance the visibility of chromosomes and improve scoring accuracy.

 16.The selected dose of 150 mg/kg body weight was chosen based on previously published studies reporting similar non-lethal and biologically effective ranges for silver nanoparticles (Kim et al., 2008; Asharani et al., 2009).“within similar rang

 17.We thank the reviewer for this valuable comment.

 We agree that the statements regarding safety and biocompatibility may have been overstated. Accordingly, the conclusions have been revised to present a more cautious interpretation of the results.

 The claims have been modified to indicate that the findings suggest potential biocompatibility under the tested conditions, rather than making definitive conclusions about safety.
